# Modulation of the intrinsic neuronal excitability by multifunctional liposomes tailored for the treatment of Alzheimer’s disease

**DOI:** 10.2147/IJN.S161563

**Published:** 2018-07-11

**Authors:** Anna Binda, Alice Panariti, Andrea Barbuti, Carmen Murano, Roberta Dal Magro, Massimo Masserini, Francesca Re, Ilaria Rivolta

**Affiliations:** 1 School of Medicine and Surgery, University of Milano-Bicocca, Monza, Italy, ilaria.rivolta@unimib.it; 2 Department of Biosciences, The PaceLab and Interuniversity Center of Molecular Medicine and Applied Biophysics (CIMMBA), University of Milan, Milano, Italy; 3 Milan Center for Neuroscience (NeuroMI), University of Milano-Bicocca, Monza, Italy, ilaria.rivolta@unimib.it; 4 Nanomedicine Center NANOMIB, University of Milano-Bicocca, Milano, Italy, ilaria.rivolta@unimib.it

**Keywords:** neurodegenerative disorders, nanomedicine, action potential, electrophysiology, patch clamp, β-amyloid peptide

## Abstract

**Purpose:**

Nanotechnologies turned out to be promising in the development of diagnostic and therapeutic approaches toward neurodegenerative disorders. However, only a very scant number of nanodevices until now proved to be effective on preclinical animal models. Although specific tests in vivo are available to assess the potential toxicity of these nanodevices on cognitive functions, those to evaluate their biosafety in vitro on neurons are still to be improved.

**Materials and methods:**

We utilized the patch-clamp technique on primary cultures of cortical neural cells isolated from neonatal rats, aiming to evaluate their electrical properties after the incubation with liposomes (mApoE-PA-LIPs), previously proved able to cross the blood–brain barrier and to be effective on mouse models of Alzheimer’s disease (AD), both in the absence and in the presence of β-amyloid peptide oligomers.

**Results:**

Data show a high degree of biocompatibility, evaluated by lactate dehydrogenase (LDH) release and MTT assay, and the lack of cellular internalization. After the incubation with mApoE-PA-LIPs, neuronal membranes show an increase in the input resistance (from 724.14±76 MΩ in untreated population to 886.06±86 MΩ in the treated one), a reduction in the rheobase current (from 29.6±3 to 24.2±3 pA in untreated and treated, respectively), and an increase of the firing frequency, consistent with an ultimate increase in intrinsic excitability. Data obtained after co-incubation of mApoE-PA-LIPs with β-amyloid peptide oligomers suggest a retention of liposome efficacy.

**Conclusion:**

These data suggest the ability of liposomes to modulate neuronal electrical properties and are compatible with the previously demonstrated amelioration of cognitive functions induced by treatment of AD mice with liposomes. We conclude that this electrophysiological approach could represent a useful tool for nanomedicine to evaluate the effect of nanoparticles on intrinsic neuronal excitability.

## Introduction

Alzheimer’s disease (AD) is one of the most studied and worldwide common neurodegenerative disorders. As such, AD has the clinical feature of a selective and progressive loss of neuronal cells, which is responsible for the typical behavioral and physical deficits. There are many different pathological events happening in the brain, such as accumulation of the β-amyloid peptide (Aβ), presence of neurofibrillary tangles of the microtubule-associated hyper-phosphorylated protein tau, neuronal and synaptic loss, cerebral atrophy, and signs of inflammation. Among these events, researchers suggest that the generation of the neurotoxic Aβ peptide from sequential amyloid precursor protein (APP) proteolysis is the crucial step in the development of AD.

So far, current different therapeutic strategies for AD offer modest and short-term benefits. Nanotechnologies, which consist in the research of tools and systems through the nanometric control of the material,^1^ are very promising in the development of both diagnostic and therapeutic approaches for neurodegenerative diseases.

Among the reasons, nanocarriers could be functionalized in order to have the ability to cross the blood–brain barrier (BBB), improving both qualitatively and quantitatively the transport of drugs directed to the central nervous system (CNS), and limiting, at the same time, side effects. In recent years, our group developed multifunctional nanoliposomes, composed of sphingomyelin (Sm) and cholesterol (Chol) and bifunctionalized with phosphatidic acid (PA) and with a peptide (mApoE) derived from the receptor-binding domain of apolipoprotein E (named mApoE-PA-LIPs) as a candidate for the treatment of AD.^2^–^4^ The presence of PA has been shown to confer to LIPs strong affinity for Aβ in different aggregation forms; mApoE-derived molecules, instead, improve the passage of nanoliposomes across the BBB either in vitro or in vivo.^5^

In vivo studies on mouse model of AD demonstrated that mApoE-PA-LIPs cross the BBB and showed the efficacy to recover long-term recognition memory and to reduce the number and total area of Aβ plaques in the brain.^6^ These same nanoliposomes have been confirmed to prevent memory loss in a presymptomatic mouse model of AD as well.^7^

The mechanism of action responsible for these improvements could be inferred by the results obtained in vitro: mApoE-PA-LIPs were able to bind to Aβ with high affinity, to inhibit the formation, and to destabilize the preformed accumulation of Aβ_1–42_ aggregates without affecting either endothelial and neuroblastoma cells’ viability or the BBB monolayer integrity. Moreover, they were internalized by cells, likely by a receptor-mediated endocytosis and transported in their intact form across the monolayer of hCMEC/D3, a model of BBB.^3^

Eventually, mApoE-PA-LIPs possess the capacity to sequester Aβ_1–42_ in human plasma and cerebrospinal fluid^4^ endorsing the hypothesis of the “sink effect” supported by the in vivo data related to the increment of Aβ in the liver and in the spleen in AD mice treated with mApoE-PA-LIPs.^6^

However, nanoparticles (NPs) used as therapeutic tool cannot be considered as an inert delivery vehicle.^8^ Even though liposomes (LIPs) are considered to be more bio-compatible with respect to other NPs, it has been reported that amine-modified polystyrene NPs or gold NPs change the membrane cellular potential in non-excitable cells upon binding, not by acting as permeabilizing agent, but by interfering with potassium channels responsible for the maintenance of the resting potential.^9^ In plant cells, silver NPs were responsible for an increase of intracellular calcium concentration that was prevented by the application of Gd^3^^+^, a calcium channel blocker; moreover, they inhibited potassium outward current as well.^10^

Since mApoE-PA-LIPs have been developed as a therapeutic tool for AD and are able to cross the BBB, attention has to be payed to their possible adverse effects. The in vivo data obtained on transgenic mice exposed to mApoE-PA-LIPs suggest that no apparent acute toxicity effect is exerted on the brain, at least under the experimental conditions adopted. Nevertheless, neurons are likely the most delicate cells of the body; thus, the possible effects on the physiological activity of excitable cells of the CNS have to be still thoroughly investigated. Indeed, the environment beyond the barrier is rich in neurons, whose electrophysiological properties should be preserved in order to maintain their electrical activity. Therefore, in this study, we evaluated the effects of mApoE-PA-LIPs on the excitable properties of primary culture of cortical neurons isolated from neonatal rats. Moreover, due to the difficulties of obtaining single neuronal cells from adult mice model of AD, we mimicked the in vivo condition incubating cortical neurons with Aβ oligomers and investigated whether mApoE-PA-LIPs retained the ability to exert their effect.

## Materials and methods

### Primary cell culture

All procedures involving animals and their care were conducted according to European and Italian laws and policies (D. Lgs. no 2014/26, 2010/63/UE) and approved by the University of Bicocca Animal Care and Use Committee that includes ad hoc members for ethical issues. Primary cultures of cortical neural cells were isolated from neonatal rats (Charles River), 3 days postbirth (P3),^11^ and cultured for 6 or 7 days (DIV6–7) in p35 dishes coated with poly-L-lysine in a controlled environment (5% CO_2_, 37°C) in Neurobasal A culture medium (Thermo Fisher Scientific, Waltham, MA, USA) supplemented with B27 (Thermo Fisher Scientific), glutamine (1 mM; Thermo Fisher Scientific), β-FGF (10 ng/mL; Thermo Fisher Scientific), penicillin G (50 U/mL) and streptomycin (50 µg/mL; Thermo Fisher Scientific). The culture contained both neurons and non-neuronal cells, such as astrocytes as the experiments of immunofluorescence proved.

The authors confirm that all the mandatory health and safety procedures have been complied with in the course of conducting any experimental work reported in the present paper.

### Preparation and characterization of multifunctional LIPs

LIPs, designed for Alzheimer’s treatment, were prepared and characterized as previously described.^12^,^13^ Briefly, LIPs were composed of a matrix of Sm and Chol (1:1 molar ratio), prepared by extrusion procedure and functionalized with PA, as Aβ-binding agent, and with a modified peptide (mApoE) derived from the receptor-binding domain of apolipoprotein E, as BBB target ligand. These multifunctional LIPs are dubbed as mApoE-PA-LIPs.

For immunofluorescence experiments, fluorescent mApoE-PA-LIPs, carrying BODIPY-FL C12-Sm (Thermo Fisher Scientific) in the lipid bilayer, were used. The fluorescent probe (0.5% mol of total lipids) was added to the lipid mixture before the extrusion procedure. To remove un-incorporated material, LIPs were diafiltered through 30,000 molecular weight (MW) cutoff membrane, as described.^6^ The yield of fluorescent probe incorporation and surface functionalization with mApoE peptide was determined by spectrofluorometric analysis. The total lipid recovery was measured by Stewart’s assay. Size and polydispersity index (PDI) were analyzed by dynamic light scattering (DLS) technique (Brookhaven Instruments Corporation, NY, USA). ζ-potential was determined by using an interferometic Doppler velocimetry with the same instrument equipped with ZetaPALS device.

### Preparation of Aβ oligomers

Aβ oligomers were prepared as previously described.^3^,^14^ Briefly, Aβ_1–42_ (Sigma-Aldrich Co., St Louis, MO, USA) lyophilized peptide was solubilized in 1,1,3,3,3-hexafluoro- 2-propanol (HFIP, Sigma-Aldrich Co) at 1 mg/mL concentration. The peptide was allowed to air dry in a chemical fume hood overnight and suspended in dimethyl sulfoxide (DMSO; Sigma-Aldrich Co) in order to obtain a peptide concentration of 5 mM. After bath sonication of 10 min, the sample was diluted to 100 µM in phosphate-buffered solution (PBS) and incubated 24 h at 4°C to obtain an oligomer-enriched preparation. The formation of oligomers was assessed by atomic force microscopy as described.^3^,^14^

### Effect of treatment with multifunctional LIPs on cell viability

Cortical neural cells were plated on 96 wells at a density of 5,000 cells/well and kept in a controlled environment (37°C and 5% CO_2_). At DIV6, cells were exposed for 4 or 48 h to the medium containing mApoE-PA-LIPs at a concentration of 10 µM. This dose was chosen based on in vivo studies as the concentration of NPs able to reach the brain.^3^,^6^ The effect of treatment with LIPs on cells’ viability was assessed by measuring the lactate dehydrogenase (LDH) release (LDH Cytotoxicity Detection KitPLUS; Hoffman-La Roche Ltd., Basel, Switzerland) and by measuring the mitochondrial enzymatic activity by MTT assay (MTT Formazan; Sigma-Aldrich Co.), as previously described.^15^ Untreated cells were used as negative control. For the LDH assay, cells lysed with a specific buffer in order to free the whole cellular LDH were used as positive control, whereas for the MTT assay, the positive control is not required.

### Cellular uptake of LIPs by immunofluorescence

Cells were seeded on glass coverslips. At the end of incubation (1, 4 or 24 h) with 10 µM of fluorescent labeled mApoE-PA-LIPs, neural cultures were fixed with paraformaldehyde (PFA), washed three times with protein-free PBS and incubated with anti-βIII Tubulin antibody (1:250; Promega Corporation, Fitchburg, WI, USA) or with glial fibrillary acidic protein (GFAP) antibody (1:400; Sigma-Aldrich Co) for 2 h at room temperature and, after washes, with Alexa Fluor 594-conjugated goat anti-mouse IgG (1:100; Thermo Fisher Scientific) for 1 h at room temperature. 1 µM DAPI (Sigma-Aldrich Co) was used to label nuclei. Images were acquired with LSM710 inverted confocal laser scanning microscope equipped with a Plan-Neofluar 63×1.4 oil objective (Carl Zeis Meditec AG, Jena, Germany). Excitation wavelengths were λ=488 nm to detect LIPs, λ=610 nm to detect βIII Tubulin or GFAP, and λ=405 nm to detect nuclei.

### LIPs–cells interaction by fluorescence imaging of live cells

Cells were seeded on glass coverslips. After 1 or 4 hours of incubation with fluorescent-labeled mApoE-PA-LIPs, neuronal cultures were washed once and medium was substituted with the extracellular solution used for electrophysiological recordings. Images were acquired with the LSM710 inverted confocal laser scanning microscope equipped with a Plan-Neofluar 63×1.4 oil objective (Carl Zeiss Meditec AG). λ=488 nm and bright field were used to detect LIPs and cells, respectively. The quantitative analysis of the green signal (mApoE-PA-LIPs) was performed using ImageJ software (ImageJ, U. S. National Institutes of Health, Bethesda, MD, USA).

### Electrophysiological recordings

Recordings on primary cortical neurons were done at room temperature (22°C–25°C) applying the patch-clamp technique in the whole-cell configuration. The set-up was equipped with a Multiclamp 700B patch-clamp amplifier, a Digidata 1440A (both from Axon Instruments, Molecular Device, Sunnyvale, CA, USA), and pClamp 10.3 software (Molecular Devices LLC, Sunnyvale, CA, USA).

The cell capacitance and the resting membrane potential were measured immediately after obtaining the whole-cell configuration. The input resistance was calculated according to Ohm’s law.

After the measurement of the passive properties in voltage-clamp mode, the amplifier was then switched to current-clamp mode, the bridge balance compensation was applied, and the membrane resting potential was held at −70 mV by injecting the appropriate current. Neuronal firing was generated injecting 1 s-long depolarizing current pulses of increasing amplitude in 10 pA steps. The minimum current necessary to elicit an action potential (AP; the so-called rheobase current) and the frequency of APs were then measured.

The internal pipette solution contained the following (in mM): 120 K-gluconate, 15 KCl, 2 MgCl_2_, 0.2 EGTA, 20 phosphocreatine-Tris, 2 ATP-Na_2_, 0.2 GTP-Na_2_, 0.1 leupeptin, and 10 K-HEPES, pH 7.2 with KOH. The bath solution contained the following (in mM): 129 NaCl, 1.25 NaH_2_PO_4_, 1.8 MgSO_4_, 1.6 CaCl_2_, 3 KCl, 10 Na-HEPES, and 5 glucose, pH 7.4 with NaOH.^16^

Recordings were carried out after the exposure of the cell cultures for 1, 4, and 24 h to mApoE-PA-LIPs at 10 µM concentration or for 4 h with LIPs functionalized only with PA (PA-LIPs) or only with mApoE (mApoE-LIPs), at 10 µM concentration. To study the effect of the presence of Aβ in the culture, cells were exposed to Aβ 500 nM for 2, 4, or 8 h. To evaluate the efficacy of the liposome treatment in presence of Aβ, we incubated the neurons with oligomers 500 nM for 8 h, the last four of which were in coincubation with 10 µM of mApoE-PA-LIPs. To note, LIPs and Aβ were not applied during the recording phase of the experiments; thus, any change observed raised from the pretreatment performed before the cell culture dish was transferred to the electrophysiological recording setup.^17^

### Statistical analyses

Data are presented as mean ± SEM. Statistical analyses were conducted using OriginPro 8 software (OriginLab Corporation, Northampton, MA, USA). Group comparisons were made with unpaired Student’s *t*-test. An ANOVA was performed for multiple comparison, followed by Fisher correction. *p*-Values of ≤0.05 were considered significant (*).

## Results

### Characterization of mApoE-PA-LIP

**Figure 1:**
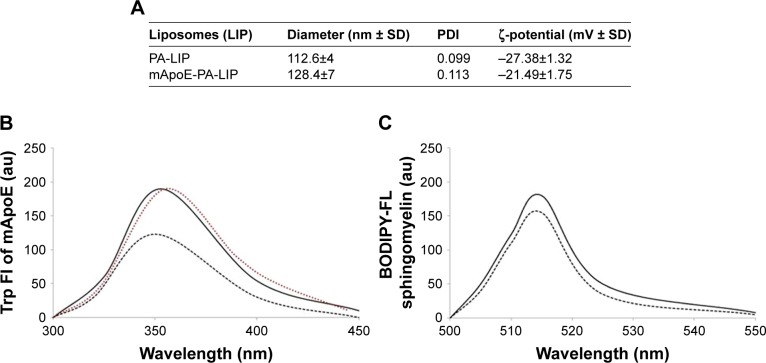
Characterization of LIPs. **Notes:** (**A**) Size, polydispersity, and ζ-potential values of PA-LIPs and mApoE-PA-LIPs determined by dynamic light scattering and interferometic Doppler velocimetry. (**B**) Fluorescent spectra of Trp-mApoE peptide in solution (red line), after incubation with LIPs (dark line) or after mApoE-PA-LIPs purification (dark dotted line). (**C**) Fluorescent spectra of BODIPY-FL C12-sphingomyelin embedded in LIPs before (−) and after purification (•••). **Abbreviations:** LIPs, liposomes; PA, phosphatidic acid.

In the present study, we utilized the previously described mApoE-PA-LIPs and PA-LIPs as a control. Size, polydispersity, and ζ-potential values are reported in Figure 1A. LIPs had a size below 200 nm diameter, low polydispersity index (PDI), and a negative ζ-potential indicating that dispersion was homogeneous in terms of dimension and was electrically stabilized. The yield of LIP surface functionalization with mApoE peptide, followed by measuring the blue shift of Trp present in the peptide sequence, was 65%±12% (Figure 1B), and the recovery of BODIPY-FL C12-Sm after LIP purification was 88.4%±9% (Figure 1C) vs a total lipid recovery >90%.

### Biocompatibility and internalization

Primary cultures of neural cells from neonatal rats were exposed to mApoE-PA-LIPs for 4 or 48 h at a concentration of 10 µM (untreated cells as control). When comparing LIPs-treated and untreated cells, LDH assay displayed a maximum of 1% of plasma membrane distress and MTT assay a 7% of decrease in the metabolic activity (Table 1). These results indicated non-significant changes in plasma membrane integrity and metabolic stress, respectively, thus a high degree of biocompatibility.

**Figure 2:**
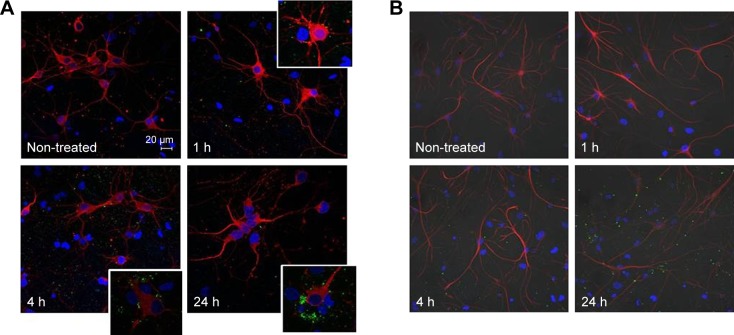
Confocal images of primary cultures of neuronal cells incubated with mApoE-PA-LIPs at different incubation time. **Notes:** (**A**) Anti-βIII tubulin antibody specifically marks neuronal cells, while (**B**) GFAP antibody specifically marks glial cells. Neural cultures were either non-treated or incubated with fluorescent mApoE-PA-LIPs for 1, 4, and 24 h. Images revealed that liposomes were not internalized by neurons (**A**) nor by glial cells (**B**). **Abbreviations:** PA, phosphatidic acid; LIPs, liposomes; GFAP, glial fibrillary acidic protein.

NPs internalization is a common phenomenon in in vitro experiments; thus, confocal laser scanning microscopy was used to observe whether fluorescent mApoE-PA-LIPs were internalized by neural cells. The images acquired after 1 h of incubation revealed no particles inside the cytoplasm of neither neurons (specifically labeled with anti-βIII tubulin antibody; Figure 2A upper panel) nor astrocytes (labeled with GFAP antibody, Figure 2B upper panel). As the incubation time was extended up to 4 and 24 h, the status of LIPs aggregation increased, and, again, the fluorescence was localized in the proximity of the cell plasmamembrane and not inside the cells (Figure 2A and B, lower panels). These qualitative data strongly suggested that neural cells did not uptake mApoE-PA-LIPs independently from the duration of their exposure.

### mApoE-PA-LIPs incubation increased neuron excitability

Since mApoE-PA-LIPs are specific NPs designed to overcome the BBB, all experiments presented from now on were conducted in order to evaluate the effects of the incubation of neurons with these given LIPs for different time intervals (1, 4, and 24 h). mApo-LIPs and PA-LIPs incubation was evaluated as a further control only when significant differences were observed in comparison to untreated cells.

Using the patch-clamp technique, we measured the passive membrane properties of neurons, such as cellular capacitance and input resistance, the resting membrane potential, the rheobase current for AP generation, and the firing frequencies against the amplitude of injected current.

Following the treatment with mApoE-PA-LIPs, cell capacitance and cell resting membrane potential did not change; however, the input resistance significantly increased by 17% in neurons incubated for 4 h with mApoE-PA-LIPs, even though an increasing trend was also present when the incubation lasted for just 1 h. Cells exposed for the same period with PA-LIPs did not show any change, while a 13% increase was observed in cells incubated with mApoE-LIPs. All these data are summarized in Table 2.

**Figure 3:**
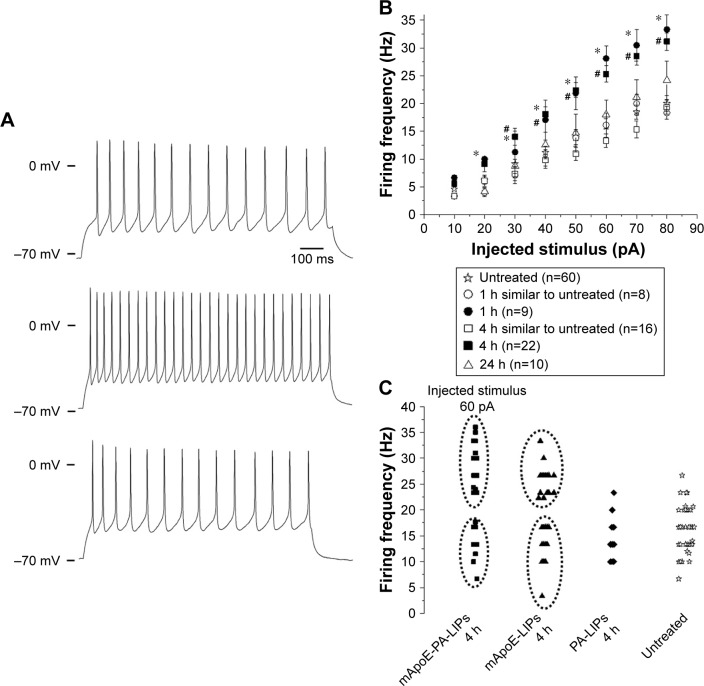
Effect of mApoE-PA-LIPs on the firing frequency of neurons. **Notes:** (**A**) Representative current-clamp recordings from neurons; APs were recorded during the application of 1 s of depolarizing injected current of 60 pA from a holding potential of −70 mV. The upper traces were recorded from untreated cells, the middle and the bottom traces were recorded from cells incubated with mApoE-LIPS for 4 h. The difference in the frequency of firing is evident. (**B**) Relationship between the average frequency of AP firing elicited and the respective amplitude of injected stimulus over the whole range of stimuli tested. Neurons incubated with mApoE-PA-LIPs for 1 h (n=17) or 4 h (n=38) displayed sub-population of cells that fired with a significantly higher frequency (filled circles and squares, respectively) compared to control condition (n=60; empty star) or to longer time incubations (n=10; empty triangles) (*,^#^*p*<0.01). For the sake of clarity of the plot, data related to the mApoE-LIPs incubation are not presented in this panel. (**C**) Scattered plot showing the existence of two populations of firing frequency in neurons incubated for 4 h with mApoE-PA-LIPs or mApoE-LIPs when the injected current was 60 pA. Untreated neurons and neurons incubated with PA-LIPs behaved similarly among themselves. **Abbreviations:** PA, phosphatidic acid; LIPs, liposomes; AP, action potential.

The minimum current necessary to elicit an AP averaged at 25.9±2 pA in untreated cells. In neurons treated with mApoE-PA-LIPs, this parameter was overall smaller; in particular, after 4 h of incubation, two cellular populations were clearly distinguishable: in about half of the cells (51%, 26 cells out of 51), the rheobase current averaged at 30.4±4 pA, while in the remaining fraction (49%, 25 cells out of 51), the rheobase current averaged at 20.4±2 pA (*p*<0.01). A very similar distribution was observed for the mApoE-LIPs incubated neurons (27.5±5 pA, n=40 and 18.06±4 pA, n=31, *p*<0.01). In the other conditions tested, all the cells started to fire when the injected current amplitude was similar to the one of the untreated cells (25.4±4, 33.3±4, and 28.6±5 for cells incubated with mApoE-PA-LIPs for 1 or 24 h and with PA-LIPs for 4 h, respectively; Table 2). Figure 3A shows representative trains of APs recorded in neurons either untreated (top) or treated with mApoE-PA-LIPs (center and bottom). Again, the analysis of the firing frequency revealed the coexistence of two different behaviors in neurons incubated for 1 or 4 h with mApoE-PA-LIPs almost at all the amplitude of current steps injected (Figure 3B). In particular, 43 and 58% of cells incubated for 1 and 4 h, respectively, exhibited a significantly higher (almost twofold) firing frequency, while in the remaining cells analyzed, the firing rate was similar to the one of untreated neurons (Figure 3C for the scattered data at 4 h of incubation). When the incubation was prolonged up to 24 h, the measured firing rate was no longer affected by the treatment.

As an example, at 60 pA of injected current, the untreated cells showed a frequency of firing of 15.9±1.4 Hz (n=60); in the population of neurons incubated with mApoE-PA-LIPs for 1 h, nine out of 16 cells (56.3%) fired at 17.1±2.4 Hz, while the remaining seven cells (43.7%) fired at 28.9±1.3 Hz (*p*<0.01). After 4 h of incubation with mApoE-PA-LIPs, 42% of cells (n=16) exhibited a frequency of 13.3±1.2 Hz and the remaining 58% (n=22) fired twice as frequently, reaching 25.3±1.5 Hz (*p*<0.01, Figure 3C). In neurons treated for 4 h with mApoE-LIPs we observed a similar behavior, with 46% of cells (26 out of 57) firing at 13.7±1.4 Hz and 54% (31 cells) firing at 23.7±0.5 Hz (*p*<0.01). In PA-LIPs, the firing frequency was 16.3±2.2 Hz in 100% of cells (n=15).

**Figure 4:**
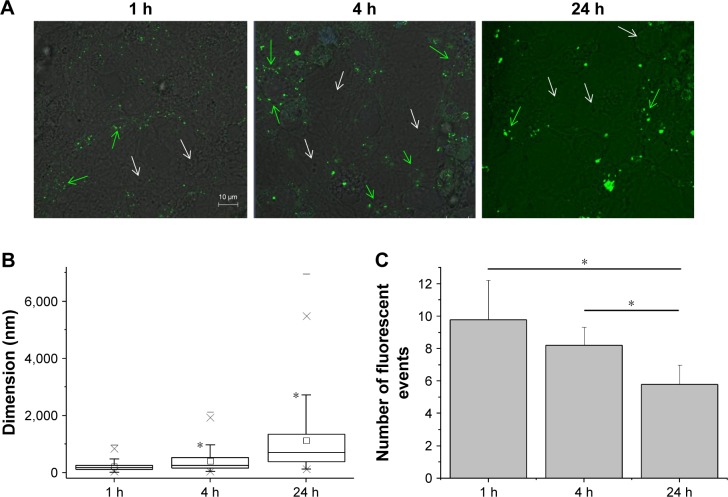
Fluorescence imaging on live cells. **Notes:** (**A**) Distribution of the fluorescent mApoE-PA-LIPs on the neuronal cell cultures after 1 h (left), 4 h (central), or 24 h (right) of incubation. Green and white arrows, respectively, indicate cells interacting and non-interacting with mApoE-PA-LIPs. (**B**) Box-chart showing the dimension of the green fluorescent spots corresponding to mApoE-PA-LIPs in the cell culture. The size increased over time (*significance versus the dimension at 1 h of incubation; *p*<0.01). (**C**) Bar graph counting, on average, the fluorescent events found in each field analyzed. **Abbreviations:** PA, phosphatidic acid; LIPs, liposomes.

To unravel the reason behind the coexistence of populations of cells firing at different frequencies, we observed the distribution of the fluorescent nanoliposomes on the neural cells cultures under similar conditions of those used for patch-clamp experiments. The differences in these live imaging pictures compared with the ones obtained with the immunofluorescence protocol (Figure 2) are related to the fact that the former samples were treated with a much more delicate approach, as described in the “Materials and methods” section, that did not include several strong washes. Figure 4A shows that while some cells were indeed in contact with mApoE-PA-LIPs (green arrows), some were not (white arrows). As well, the fluorescent spots (ie, mApoE-PA-LIPs) over the culture layer significantly increased their dimension with time, being 214±63, 403±153, and 1,125±488 nm at 1, 4, or 24 h of incubation, respectively (Figure 4B). Moreover, the amount of cells having mApoE-PA-LIPs adherent to their surface was higher after 1 or 4 h of incubation and decrease at 24 h; in fact, the number of fluorescent events counted per field with time was 9.78±1.01, 8.2±0.46 and 5.8±0.5 at 1, 4 or 24 h of incubation, respectively (Figure 4C). Unfortunately, for technical reasons, we could not perform patch-clamp experiments using fluorescent mApoE-PA-LIPs.

### Effects of Aβ oligomers incubation or Aβ oligomers and mApoE-PA-LIPs co-incubation on neuron properties

**Figure 5:**
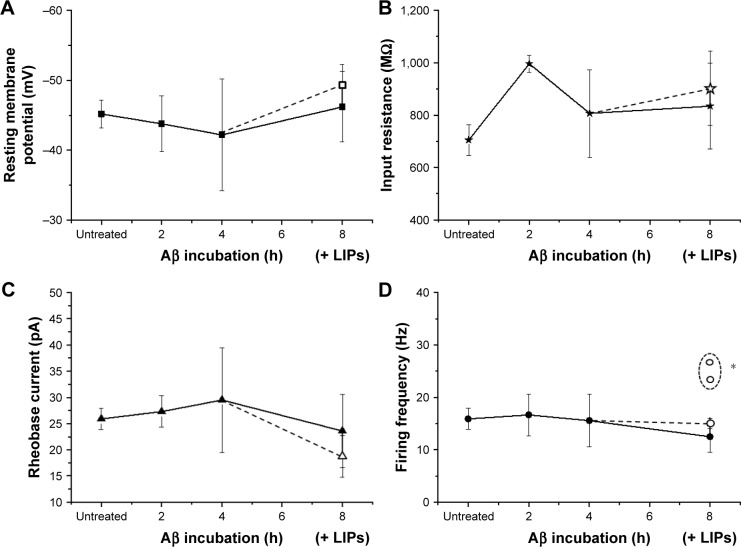
Effects of Aβ oligomers or Aβ oligomers and mApoE-PA-LIPs coincubation on neuron properties. **Notes:** Panels represent the resting membrane potential (**A**), the cell input resistance (**B**), the rheobase current (**C**), and the firing frequency (**D**) in untreated neurons and in neurons incubated with Aβ oligomers for 2, 4, or 8 h (data connected with the straight line). The empty symbols represent the same parameters measured after 4 h of Aβ incubation alone followed by 4 h of coincubation with Aβ plus mApoE-PA-LIPs. (**D**) The empty circles are reported as averaged value of four out of six cells firing as the untreated ones, while the two empty circles in the dashed profile represent the subpopulation of neurons (two out of six) firing at a significantly higher frequency (*p<0.01). **Abbreviations:** Aβ, β-amyloid peptide; PA, phosphatidic acid; LIPs, liposomes.

In order to study the properties of neurons in presence of amyloid beta, we incubated the neural cultures with 500 nM of Aβ oligomers for 2, 4, and 8 h. The treatment had no effect on the neuronal resting membrane potential (−43.85±4 mV, 2 h, n=5; −42.21±8 mV, 4 h, n=18; and −46.2±5 mV, 8 h, n=15), on the cell input resistance (996±32 MΩ, 2 h, n=4; 806±167 MΩ, 4 h, n=15; and 834±164 MΩ, 8 h, n=13), on the rheobase current (27.3±3 pA, 2 h, n=4; 29.5±10 pA, 4 h, n=19; and 23.6±7 pA, 8 h, n=14), and on the frequency of firing (16.6±4 Hz, 2 h, n=3; 15.5±5 Hz, 4 h, n=6; and 12.5±4, 8 h, n=8) (Figure 5A–D). At last, in order to test the efficacy of mApoE-PA-LIP on neuronal excitability in presence of Aβ in the culture, after 4 h of incubation with the oligomers, mApoE-PA-LIPs were added for 4 h (ie, 4 h of incubation of Aβ alone plus 4 h of coincubation of Aβ and mApoE-PA-LIPs). Under this condition, the cell resting membrane potential, the input resistance, and the rheobase current measured did not change significantly (−49.3±3 mV [n=9], 902±141 MΩ [n=8] and 18.7±4 pA [n=8], respectively). Interestingly, though, concerning the frequency of firing, we could still point out the presence of two populations of neurons: one firing at 15±1 Hz (n=4) and another composed by two cells at 23.3 and 26.7 Hz, respectively (Figure 5D).

## Discussion

The main obstacles to the development of potential therapeutic approaches for neurodegenerative diseases are the anatomical and physiological organization of the BBB, which is a weir of endothelial cells surrounding the capillaries of the CNS, preventing the passage of almost all pharmaceuticals and, thus, protecting the brain.

In the past few years, it has been proven that appropriately functionalized NPs are able to cross the BBB, therefore representing a potential diagnostic and therapeutic tool of considerable impact in neurodegenerative disorders. In this context, a prominent place is certainly occupied by NPs developed for the treatment of AD, one of the most common causes of dementia in the world. AD is responsible for a progressive and irreversible loss of neuronal cells that, in turn, involves the manifestation and the sharpening of cognitive, behavioral, and physical deficits.

Several studies have previously suggested that mApoE-PA-LIPs could potentially be advantageously used with regard to AD as they proved to reduce the Aβ burden in mouse animal models of the disease and to prevent memory impairment in a model of presymptomatic stage of AD.^6^,^7^ This capability is likely attributable to the presence, on their surface, of PA molecules conferring the ability to bind Aβ with high affinity^2^ and of the peptide containing the receptor-binding sequence of ApoE, improving their transport through the BBB.^13^

In the present study, we evaluated the effects of these double functionalized LIPs on primary cultures of cortical neural cells isolated from neonatal rats with a particular attention to the neuron electrophysiological activity.

Previous results demonstrated the biocompatibility of mApoE-PA-LIPs on hCMEC/D3 cells,^3^ on HUVEC cells and on macrophages RAW264.7.^18^ Our data demonstrated that mApoE-PA-LIPs were biocompatible also with neural cells, as we did not detect signs of distress neither on the plasmamembrane (LDH release) nor on the metabolic activity (MTT production) for up to 48 h of incubation. This is an important result considering that mApoE-PA-LIPs role is likely to disaggregate and bind Aβ within the brain parenchyma.

Interestingly and differently to what has been previously observed in other cellular systems,^3^ these LIPs were not internalized in neurons nor in astrocytes. Low-density lipoprotein receptor (LDLR) and LDLR-related protein 1 (LRP1) are the main metabolic receptors for lipoproteins in the brain, the first being mainly expressed in the astrocytes and the latter most expressed in neurons and in particular in cortical neurons of neonatal rats.^19^ Upon binding ApoE, LDLR and LRP1 form endocytotic vesicles to transport their ligands from the cell surface to intracellular compartments.^20^ From our data, the internalization of the mApoE-PA-LIPs was prevented in neural cells. As already stated, LIPs used in this study exhibit the motif constituted by residues 141–150 of human apolipoprotein E. This motif possesses the conserved lysines and arginines crucial for the interaction between ApoE and LDLRs and subsequent nanoliposome internalization in endothelial cells.^3^ Studies, not related to NPs, showed that in fibroblasts, the minimal sequence of ApoE recognized by the receptor has to be bound to lipoprotein in order to allow the internalization of the complex ligand–receptor,^21^ and in HepG2 cells, it has to be linked to a class A amphipathic peptide.^22^ Up to now, there is no information regarding the internalization of the complex ligand–receptor in neurons, when only the minimal sequence of ApoE is present for the binding. Indeed the VLDL receptor does not require the association of ApoE with lipids for recognition and binding, whereas the LRPR prefers lipid-bound forms of ApoE.^23^ In order to unravel the reason why LIPs are not internalized by neurons, one may incubate these cells with a series of synthetic sequences of ApoE of different length to study if there are specific requirements in the apolipoprotein E sequence for the internalization in this system. Yet, this is far beyond the goal of this paper.

The study of the electrical properties of the cells after mApoE-PA-LIPs incubation showed no changes in neuronal cell capacitance, but a significant increase in their input resistance. Considering that mApoE-PA-LIPs are primarily composed of lipids, that in electrical terms represent elements increasing the membrane electrical isolation, we can hypothesize that if LIPs were not fused with the cell membrane (as Figures 2 and 5 suggest), they only adhered or promoted a minor plasmalemmal lipids incorporation, thus justifying the lack of the effects on the capacitance of the cell. This may however bring to either a redistribution of charge along the surface of the membrane of the excitable cells and thus the resistance may be altered^24^,^25^ or a direct effect of the lipids on the ion channels gating. In fact, while neutral lipids (ie, phosphatidylethanolamine, phosphatidylcholine) have little effect on channel activity, lipids with highly negatively charged head groups (ie, PA) strongly modulate ion channels.^26^ Data obtained incubating neurons with mApoE-PA-LIPs were comparable with those obtained with mApoE-LIPs (thus without PA and its negatively charged head groups) leaning toward the first hypothesis. Moreover, we hypothesize that the reason why cells exposed to PA-LIPs did not show any change in input resistance compared to mApoE-PA-LIPs is related to the fact that without mApoE, the liposome may not stick to the cell membrane.

The value of the resting membrane potential was not different after incubation of neurons with mApoE-PA-LIPs, while the rheobase current decreased significantly, coherently with the increase in the input resistance. The same results were obtained when the incubation was done with mApoE-LIPs.

In terms of firing frequency, in neurons incubated with mApoE-PA-LIPs (as well as with mApoE-LIPs) for a period up to 4 h, we could identify two different populations: one population that fired like the untreated cells and a second population that fired at twice that frequency. Longer incubation periods resulted in the disappearance of this latter population of cells. Taking advantage of the live fluorescence images, we can speculate that the mApoE-PA-LIPs dispersed in the culture medium had the (static) chance to interact with cultured neurons. But, over time, they aggregate as evidenced by the increase in the area of the single fluorescent spot. By aggregating, the number of objects available to interact with the cells decreased, as demonstrated by the number of fluorescent events counted per field, making the interaction less probable, thus detectable. From the data shown, it seems that the ideal time to observe the interaction phenomenon and its consequences on neurons is between 1 and 4 h of incubation. At 24 h, the aggregation phenomenon seized the LIPs limiting the number of the neuron-mApoE-PA-LIPs interaction events; and in fact, after 24 h of incubation with mApoE-PA-LIPs, the effect on firing rate was not distinguishable from that of the untreated or PA-LIPs treated neurons.

Overall, the results obtained after the incubation of neurons with mApoE-PA-LIPs were very similar to the ones gathered after mApoE-LIPs incubation, suggesting that mApoE peptide is mainly responsible for the changes observed in the electrical properties of the cells.

Since AD implies the presence of Aβ oligomers and plaques in the brain, it would be interesting to have the possibility to investigate the efficacy of mApoE-PA-LIPs on the electrical properties of neurons isolated from an AD model. The literature reports few studies in which this topic was investigated in vivo,^27^ or in hippocampal slices, or in primary hippocampal neurons from neonatal mice.^17^,^28^ Results demonstrated a hyperactivity in terms of calcium release^27^ and in the increase in the firing frequency of APs evoked in response to current steps^28^ leaving many basic intrinsic neuronal properties unaltered.^17^,^28^ Indeed, the isolation of cortical neurons from adult rats or mice is not easily feasible. In the literature, there is only one paper aiming at developing a method for preparing isolated cells of medial vestibular nucleus (MVN) from adult mice, which however remain viable only for 3 days in culture.^29^ These cells, however, are not consistent with our model. Moreover, in order to study the electrical properties of isolated neurons, embryonic or neonatal animals are preferred for culturing. For these reasons, we mimicked the AD model, treating the primary neural cultures with Aβ oligomers^17^ for increasing time of incubation. Consistent with previous results,^17^,^28^ also in our study, Aβ did not induce changes in the passive properties of neurons, and also the firing frequency remained stable, as reported previously.^17^ The reason why we did not detect hyperactivity in neurons incubated with Aβ may simply rely in the fact that we recorded APs in single cells and not in brain slices. Moreover, the strategy we used for the incubation is a balance between a reasonable time to allow Aβ to produce its effects and the inevitable decline that it produces with the inevitable decline on cell viability that it produces with time, which may not allow patch-clamp experiments to be performed at all. It is worthy of note, however, that after the incubation with Aβ alone followed by 4 h of co-incubation of Aβ plus mApoE-PA-LIPs, we could still detect a fraction (2 cells out of 6) of the neurons firing at higher frequency compared to the neurons incubated for 8 h only with Aβ, suggesting that the efficacy of mApoE-PA-LIPs is maintained even in presence of the oligomers.

## Conclusion

The most relevant results concerning the effect of the incubation of neurons with mApoE-PA-LIPs were the transient increase in the input resistance and the decrease of the rheobase current, consistent with an ultimately transient increase in neuronal excitability. In particular, a higher input resistance means that less synaptic input (or current injection) is necessary to lead to larger voltage changes, as the decrease in the rheobase current confirmed. Thus, changes in input resistance can change the responsiveness of the neurons to other inputs. This adjustment in intrinsic excitability is usually at the basis of plasticity of the response of the membrane of excitable neurons acting as a metaplasticity mechanism by lowering the threshold for synaptic changes.^30^

Metaplasticity develops as a result of a series of time-dependent events: an initial priming event (eg, LIPs adhesion) induces physiological or biochemical changes in neurons that can modulate plasticity induced by a subsequent event (eg, electrical stimulation); these changes may have an impact, for example, in the enhanced ability of an excitatory postsynaptic potential (EPSP) to generate an AP, again, as proved by the decrease in the rheobase current found.^31^

Modulation of intrinsic excitability may act as an adjustable gain control mechanism and have consequences for the integration processes.

Aging-related deficits in learning have been linked to failure to modulate intrinsic excitability, in the sense that a reduced intrinsic excitability may be an important predictor of cognitive decline; with this in mind, we may argue that an increase in intrinsic excitability, as we found after the incubation of neurons with mApoE-PA-LIPs, may be a predictor of cognitive preservation.

However, since the changes in intrinsic plasticity were transient, we cannot say that they code for the memory itself, but this transient enhancement of excitability may promote processes that allow successful memory formation and that facilitate acquisition of new learning.

It is well known that alterations in intrinsic properties of neuronal membranes are regarded as an important mechanism involved in the abnormal excitability of cortical circuitry and possibly account for the susceptibility to initiation and spread of seizures.^30^,^32^,^33^ Nevertheless, in a previous study where mice were treated with mAPO-PA-LIPs for 7 months and observed for 3 more months,^7^ no seizure-related behaviors such as immobility, compulsive spitting, alteration in locomotor activity, social relationships, and reactivity to environmental stimuli^34^ were observed.

LIPs and, in particular, mApoE-PA-LIPs have great potential in biotechnology and medicine, in particular in the treatment of AD. Some of the initial concerns regarding their possible toxicity have been widely alleviated by the demonstration of their biocompatibility in several cellular models as well as in primary neural cultures. Their ability to reduce Aβ accumulation in the brain^6^ brought us to investigate their effect on electrical properties of neurons, given their proximity when passing the BBB. Data presented here, besides adding some relevant information on the lack of LIPs internalization by neuronal cells, indicate that treatment with mApoE-PA-LIPs transiently increases neuronal excitability and suggests the basis for the interpretation of the cognitive recovery found in previous work.^7^ The modulation of the intrinsic excitability could be an important factor in the search for neurobiological approaches to mitigate or prevent the onset of aging-related cognitive impairments and even rescue those deficits after they emerge.

## Figures and Tables

**Table 1:** Biocompatibility of PA-LIPs and mApoE-PA-LIPs

LIPs	Exposure time (h)	LDH release (%)	Reduction in MTT cleavage (%)
Untreated	4	0±3.5	0±1.12
mApoE-PA-LIPs	4	0.5±2.1 (*p*=0.5)	3.2±1.3 (*p*=0.2)
Untreated	48	0±1.5	0±1.4
mApoE-PA-LIPs	48	0.14±1 (*p*=0.6)	4±0.5 (*p*=0.3)

**Notes:** The release of LDH represents a sign of membrane damage, while the reduction in the cleavage of MTT to obtain formazan is an index of a decrease in the metabolic activity. Results (presented as mean ± SE) are representative for three independent experiments (n=4 for each experiment); *p*-value is calculated vs non-treated cells.

**Abbreviations:** PA, phosphatidic acid; LIPs, liposomes; LDH, lactate dehydrogenase.

**Table 2:** Passive membrane properties and other parameters measured in untreated neurons or in neurons incubated with LIPs

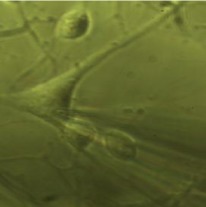	Untreated (n=73)	mApoE-PA-LIPs (1 h) (n=17)	mApoE-PA-LIPs (4 h) (n=51)	mApoE-PA-LIPs (24 h) (n=9)	PA-LIPs (4 h) (n=15)	mApoE-LIPs (4 h) (n=71)
Cell capacitance (pF)	24.89±2	27.89±2	27.18±2	29.95±3	30.67±4	24.40±2
Resting potential (mV)	−45.25±2	−47.67±4	−45.46±2	−45.01±2	−50.20±3	−49.51±3
Input resistance (MΩ)	704±58	821.71±103	822±102 (*p*=0.04)	661.79±108	638.72±77	795±126 (*p*=0.04)
Rheobase current (pA)	25.9±2	25.4±4	30.4±4 (n=25) 20.4±2 (n=26) (*p*<0.01)	33.3±4	28.6±5	27.5±5 (n=40) 18.06±4 (n=31) (*p*<0.01)

**Notes:** Data are presented as mean ± SEM and are representative of at least three independent experiments. In the inset, an image of a typical neuron under patch-clamp experiment.

**Abbreviations:** LIPs, liposomes; PA, phosphatidic acid; SEM, standard error of the mean.
